# Predicting driver fatigue using HRV measures and machine learning

**DOI:** 10.3389/fspor.2026.1796180

**Published:** 2026-06-03

**Authors:** Maya Arlini Puspasari, Annisa Marlin Masbar Rus, Danu Hadi Syaifullah, Richard Joseph Hanowski, Hasna Hamida Nurkamila, Layla Maulidina Ghaisani, Yosephine Anastasia Pardede, Dinda Anggina Aviani

**Affiliations:** 1 Department of Industrial Engineering, Faculty of Engineering, Universitas Indonesia, Depok, Indonesia; 2 Motorcycle Safety Solutions, LLC, Blacksburg, VA, United States

**Keywords:** driver fatigue, driving duration, ensemble learning, fatigue detection, heart rate variability, machine learning, sleep deprivation

## Abstract

Driver fatigue is a critical factor in traffic accidents and is strongly influenced by sleep deprivation and prolonged driving. This study examines physiological and subjective fatigue responses by combining Heart Rate Variability (HRV) indicators with the Karolinska Sleepiness Scale (KSS) and Rating of Fatigue (ROF) in a controlled driving simulation. Forty participants completed four driving sessions under normal sleep and sleep-deprived conditions, with HRV recorded in each session. The results show that increased driving duration was associated with autonomic imbalance, as reflected in lower mean RR, mean HR, RMSSD, and HF, which were associated with increased fatigue levels. In contrast, sleep duration did not significantly affect most HRV indices, although it had robust effects on subjective fatigue measures (ROF and KSS). Significant correlations were observed between HRV parameters and ROF, indicating that physiological changes aligned with subjective perceptions. To complement the statistical analysis, Logistic Regression and ensemble learning models were applied to classify fatigue-related conditions. XGBoost achieved the highest performance, with an accuracy of 77%, and identified Mean RR, mean HR, and LF as the most influential predictors. These findings indicate that combining HRV metrics with machine learning improves fatigue pattern detection and offers strong potential for developing real-time fatigue monitoring systems. The study highlights the importance of integrating objective and subjective measures to evaluate driver fatigue, particularly in conditions of sleep restriction and extended driving duration.

## 1 Introduction

Fatigue is a mental and physical condition that significantly reduces an individual's alertness, attention, and ability to complete tasks safely and effectively. The central nervous system regulates it through sympathetic and parasympathetic pathways, and it manifests as drowsiness, decreased concentration, slower reaction times, and impaired decision-making (1–4). In transportation, driver fatigue remains a significant safety concern. Reports from the National Transportation Safety Committee (KNKT) indicate that fatigue contributes to a substantial proportion of traffic accidents in Indonesia, accounting for up to 60 percent of total cases (5). Recent data from the Indonesian National Police (2023) also reported more than 148,000 road accidents, the highest in five years, indicating the severity of fatigue-related risks (6). This Indonesian context is particularly concerning, as studies indicate that 61% of traffic accidents involve human factors related to driver skill and character, with fatigue and drowsiness among the leading contributors (7). The prevalence of drowsy driving in Indonesia is alarmingly high: 79% of drivers report at least one episode of drowsy driving, and 32% experience near-miss accidents while drowsy (8).

Multiple interconnected factors, including sleep deprivation, prolonged driving, monotonous driving environments, and psychological workload, contribute to driver fatigue (9–11). The relationship between insufficient sleep and impaired driving performance is well established in prior studies. Sleep deprivation (sleeping less than 4 h) substantially increases the likelihood of sleepy driving compared to the recommended 7–8 h per night, with drivers who sleep less than 4 h facing an 11.5-fold increase in crash odds (11). Driving after 17–18 h of continuous wakefulness impairs performance to the level of a 0.05% blood alcohol concentration, and prolonged driving beyond 2 h without rest is associated with impaired motor coordination and increased microsleep episodes (12). highlights that ocular indicators, such as saccadic velocity and blink duration, along with physiological indicators, such as increased alpha and theta EEG power, are among the most reliable measures of impaired driving performance under sleep-deprived conditions. These findings underscore the importance of understanding both the physiological and psychological mechanisms underlying fatigue development.

Fatigue can be categorized into cognitive fatigue, which arises from sustained cognitive demands (13), and sleep-deprived fatigue, which results from insufficient sleep and is associated with stronger physiological dysregulation (14). Compared to cognitive fatigue, sleep-deprived fatigue tends to show more pronounced and consistent changes in physiological indicators, making it more detectable using objective measures (15).

Subjective measures such as the Karolinska Sleepiness Scale (KSS) and Rating of Fatigue (ROF) have been widely used to capture perceived alertness. While practical, subjective assessments are susceptible to misreporting because individuals may misjudge or underestimate their fatigue levels (16, 17). To address this limitation, recent studies have increasingly adopted objective physiological indicators, particularly Heart Rate Variability (HRV). HRV reflects autonomic balance and has been used as a biomarker for stress, fatigue, and changes in physiological regulation (18, 19). Reductions in HRV are commonly observed during fatigue (20), and advances in wearable sensors enable precise, non-invasive HRV monitoring. HRV parameters can be divided into time-domain (e.g., SDNN, RMSSD) and frequency-domain (e.g., LF, HF, LF/HF) measures. Previous studies have shown that time-domain HRV parameters are more reliable than frequency-domain parameters (21–23).

A comprehensive systematic review of 18 studies examining HRV-based driver fatigue detection found that, while HRV shows promise as a fatigue indicator, detection accuracy varied substantially across studies, ranging from 44% to 100%, highlighting the need for more robust, standardized methodologies (24). The inconsistency in results can be attributed to differences in experimental protocols, fatigue-induction methods, HRV feature-extraction approaches, and classification algorithms. Recent technological advancements in wearable sensors have made HRV monitoring increasingly feasible in real-world driving contexts, enabling precise, non-invasive, continuous assessment. Studies have demonstrated that HRV features extracted from as little as 2 min ECG signals can achieve fatigue detection accuracy exceeding 90% (25).

Although HRV has been explored in fatigue research, most studies have been conducted in occupational settings such as aviation, manufacturing, and healthcare (26). Research on driver fatigue in the Indonesian context, particularly systematic comparisons between sleep-deprived and normal-sleep conditions under prolonged driving, remains limited. In addition, prior studies predominantly employed conventional statistical or single-model machine learning approaches such as logistic regression, support vector machines, or decision trees. These models often have limited capacity to capture nonlinear interactions in physiological signals.

To address these gaps, this study employs ensemble learning methods, specifically Random Forest and XGBoost, to analyze fatigue patterns derived from multimodal data combining HRV parameters and subjective fatigue indicators. Ensemble methods offer substantial advantages over single-model approaches by aggregating predictions from multiple base learners, thereby improving classification accuracy, reducing the risk of overfitting, and enhancing model generalization to unseen data. Random Forest, as a bagging ensemble technique, constructs multiple decision trees trained on bootstrapped samples and averages their predictions, providing robust performance even with high-dimensional feature spaces (27). XGBoost, an advanced gradient boosting implementation, builds trees sequentially, with each new tree attempting to correct errors made by previously trained trees, and incorporates regularization techniques to prevent overfitting (28). Recent applications of ensemble learning across various domains have demonstrated superior performance over conventional methods, with XGBoost frequently achieving state-of-the-art results in machine learning competitions and practical settings (29).

This study incorporates ensemble learning methods to analyze fatigue patterns derived from HRV and subjective indicators. Ensemble models, such as Random Forests and XGBoost, provide improved accuracy by aggregating multiple learners and can identify complex relationships among features (30). These models also allow systematic evaluation of feature importance, enabling researchers to determine which HRV parameters contribute most strongly to fatigue classification. While HRV, subjective fatigue measures, and machine learning have been widely studied, this study uniquely integrates these approaches within a controlled experimental design that simultaneously examines the combined effects of sleep deprivation and prolonged driving duration. In addition, this study incorporates SHAP-based interpretability to evaluate the relative sensitivity of HRV features, providing deeper physiological insights beyond conventional classification approaches. The integration of sleep duration, driving duration, HRV data, and ensemble-based modeling has not been extensively examined in previous studies, making this approach methodologically relevant and novel.

Therefore, this study aims to analyze the effects of different sleep durations and driving durations on driver fatigue by combining objective HRV data and subjective measures (KSS and ROF) in a controlled driving simulation. This study hypothesizes that sleep deprivation and prolonged driving duration will significantly increase driver fatigue, as reflected in both HRV parameters and subjective measures (KSS and ROF). The expected outcomes are to identify physiological indicators associated with variations in sleep and driving duration, strengthen empirical evidence for fatigue assessment frameworks, and support the development of HRV-based fatigue-monitoring and alert systems informed by ensemble learning.

## 2 Materials and methods

### 2.1 Participants

This experimental quantitative study employed a within-subjects design to assess the effects of sleep duration and driving duration on driver fatigue. A total of 40 participants (aged 18–25 years; mean age = 21.3 ± 1.9 years) were recruited through purposive sampling. All participants held a valid driving license and had at least one year of driving experience.

The inclusion criteria were:

1. Healthy individuals with no cardiovascular or neurological disorders,
2. Not under the influence of caffeine, alcohol, nicotine, or medication within 24 h before data collection,
3. Normal or corrected-to-normal vision, and
4. Having a regular sleep schedule.

Participants who were pregnant, had sleep disorders, or failed to comply with pre-experiment protocols were excluded. Ethical approval was obtained from the Universitas Indonesia Research Ethics Committee, and written informed consent was collected from all participants before the study in accordance with the Declaration of Helsinki.

The adequacy of the sample size was verified using G*Power 3.1, with *α* = 0.05 and an effect size (f) of 0.25, resulting in a statistical power (1−*β*) of 0.87, exceeding the recommended minimum of 0.80. This confirmed that the number of participants was sufficient to detect significant effects.

### 2.2 Experimental design and procedure

**Figure 1:**
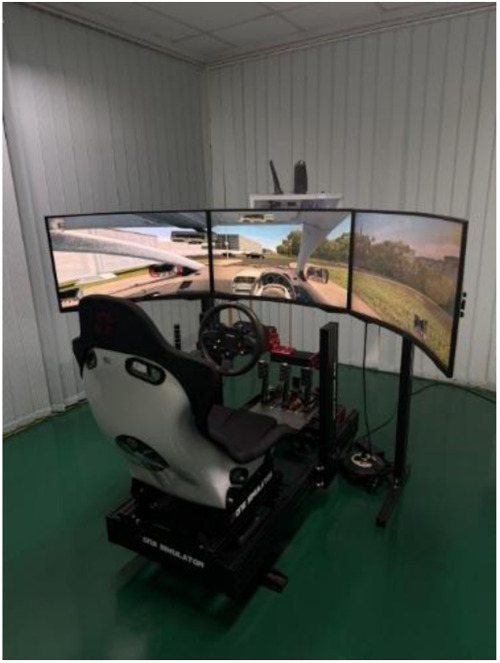
The driving simulator used in this study.

The experiment was conducted in a driving simulation laboratory designed to replicate real-world driving conditions in a controlled environment. Data were collected using a high-fidelity driving simulator from One Simulator located in the Ergonomics Centre laboratory. The simulator used three screens and controls that resembled a real car, replicating real-world vehicle dynamics and traffic scenarios. Environmental conditions were also standardized; the room temperature was maintained at 22°C, ambient noise was kept below 40 dB, and all sessions were conducted between 13.00 PM and 16:00 PM to maintain consistent circadian effects. These controls ensured consistency across participants and reproducibility of the experimental setup. Figure 1 shows the driving simulator used in this study.

Two primary experimental conditions were tested, which were (1) Normal Sleep Condition (≥7 h of sleep) and (2) Sleep-Deprived Condition (≤4 h of sleep). Participants were instructed to maintain their usual sleep schedule during the normal sleep condition and to restrict their sleep to no more than four hours for the deprivation condition. Each condition was conducted at least 48 h apart to minimize carry-over effects. The sleep duration for each participant was monitored using Fitbit smartwatches (Fitbit Inspire 2, USA) to ensure their compliance.

Upon arrival at the laboratory, participants completed a short demographic and health questionnaire. During the driving task, participants were exposed to a standardized road simulation environment with consistent weather, lighting, and traffic density to control external variability. Each participant completed four 30 min driving sessions. They wore the Polar H10 during every driving session; however, HRV data were recorded for 5 min at the end of each session. The decision to record HRV for only 5 min at the end of the driving task was made to minimize potential artifacts and distractions during the driving simulation. Continuous HRV monitoring could have interfered with the ecological validity of the driving experience, as participants might have been more aware of the monitoring equipment.

**Figure 2:**
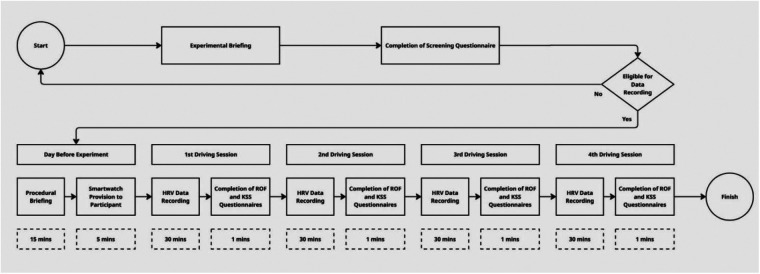
Experiment procedure.

Immediately after the driving task, participants completed two subjective fatigue questionnaires, the Karolinska Sleepiness Scale (KSS) and the Rating of Fatigue (ROF), to assess perceived drowsiness and fatigue. Each experimental session lasted approximately 120 min, including preparation and data collection. A trained research team supervised all experimental procedures to ensure safety, standardization, and adherence to the research protocol. Figure 2 shows the experiment procedure.

### 2.3 Data recording

#### 2.3.1 Heart rate variability (HRV)

HRV data were recorded using a Polar H10 heart rate sensor placed on the participant's chest. The recorded data were exported to Kubios HRV Scientific (version 3.6) for analysis. ECG signals were sampled at 130 Hz. HRV was analyzed in 5 min segments, consistent with standard short-term HRV protocols. R-peaks were detected using Kubios' built-in QRS detection algorithm, which is based on the Pan-Tompkins method. Artifact correction was performed using Kubios' automatic artifact removal tools to ensure signal validity. The 5 min HRV segments were recorded at the end of each driving session, so each participant had 4 data sets.

Data preprocessing included artifact correction (using a threshold-based filter) and interpolation to remove noise and ensure accurate HRV computation. The following HRV parameters were extracted:

1. Time-domain features: Mean RR (mean of RR intervals), SDNN (standard deviation of RR intervals), RMSSD (root mean square of successive differences), and Mean HR (mean of Heart rate).
2. Frequency-domain features: VLF (Very Low Frequency), LF (Low Frequency), HF (High Frequency), and LF/HF ratio, calculated using Fast Fourier Transform (FFT).

#### 2.3.2 Subjective fatigue assessment

Subjective fatigue levels were assessed using two validated scales:

1. Karolinska Sleepiness Scale (KSS): a 9-point Likert scale where one represents “very alert” and nine represents “very sleepy,” used to assess perceived sleepiness.
2. Rating of Fatigue (ROF): an 11-point Likert scale (0 = not fatigued, 10 = extremely fatigued) designed to quantify perceived fatigue intensity.

Both instruments were administered immediately after each driving session. The combination of KSS and ROF enabled analysis of subjective fatigue dynamics across varying sleep and driving durations.

### 2.4 Data analysis

Repeated Measures ANOVA was conducted to examine the effects of sleep duration and driving duration on the dependent variables. The dependent variables were HRV parameters (Mean RR, SDNN, Mean HR, RMSSD, VLF, LF, HF, and LF/HF) and subjective fatigue indices (ROF and KSS). The Spearman's rank correlation was used to evaluate associations between HRV parameters and subjective fatigue indices.

**Figure 3:**
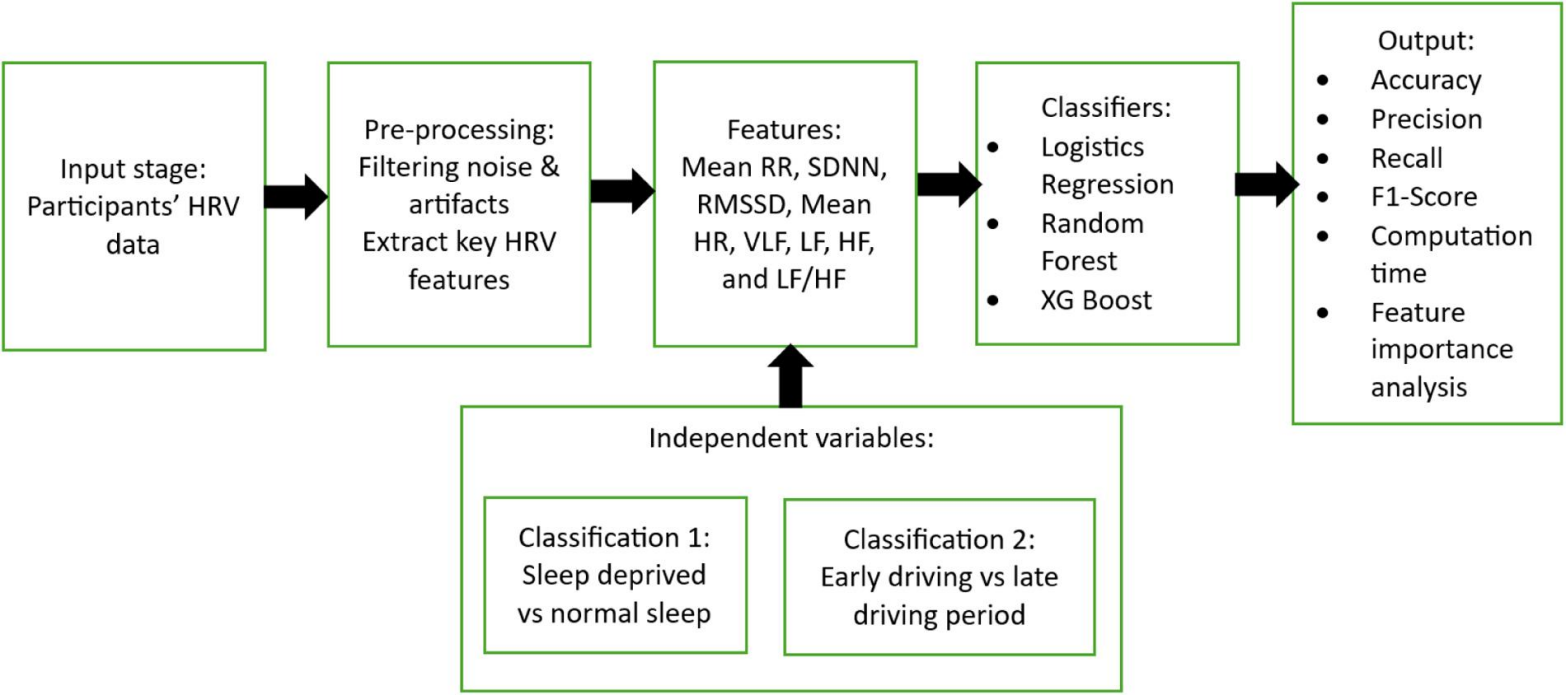
Data classification process.

In addition to conventional statistical analysis, machine learning methods were used to classify fatigue-related conditions based on HRV and subjective measures. Logistic Regression served as the baseline model. Ensemble learning models, including Random Forest and XGBoost, were then applied to capture nonlinear relationships across features and to evaluate the predictive performance of multivariate indicators. These models were also used to assess feature importance and identify the HRV parameters that contributed most strongly to fatigue classification. Figure 3 shows the data classification process.

A machine learning model in Python and Jupyter Notebook 6.5.4 was developed using supervised learning. The first step in data preprocessing was to remove noise and artifacts from the raw data. The second step was to extract key HRV features for analysis. The selected columns were based on heart rate variability metrics, such as Mean RR, SDNN, Mean HR, RMSSD, VLF, LF, HF, and LF/HF. We classified fatigue using operational definitions: periods of sleep deprivation and late driving were considered to pose a high risk of fatigue, whereas periods of normal sleep and early driving were considered to pose a lower risk of fatigue (11, 31, 32). This study employed two classifications: sleep-deprived vs. normal sleep and early driving period vs. late driving period. The sleep-deprived and late driving period (sessions 3 and 4) was categorized as fatigued (label: 1), and the normal sleep and early driving period (sessions 1 and 2) were categorized as non-fatigued (label: 0) (33). The data were split into training (70%) and test (30%) sets. All results were interpreted to assess the extent to which sleep duration and driving duration influenced both objective HRV metrics and subjective fatigue ratings, and to evaluate the ability of machine learning models to differentiate fatigue-related conditions.

The models were employed to classify fatigue states using HRV-derived features under a subject-independent evaluation framework. To prevent information leakage arising from repeated measurements within individuals, all data partitioning was performed using Group k-Fold cross-validation, with participant identifiers treated as grouping variables. Hyperparameter optimization was conducted via randomized search over a predefined parameter space, including tree depth (a random integer between 2 and 6), learning rate, number of estimators (a random integer between 100 and 200), subsampling ratios, and regularization terms. The optimization procedure used 5-fold Group k-Fold cross-validation to maximize the area under the receiver operating characteristic curve (AUC). The resulting optimal hyperparameters were subsequently fixed and reused in downstream analyses to avoid bias and reduce computational overhead.

Model performance and robustness were further evaluated using subject-level bootstrap resampling with 1,000 iterations; however, Random Forest was limited to 500 iterations due to its computational inefficiency. In each bootstrap iteration, participants were sampled with replacement, and all corresponding observations were retained to preserve intra-subject dependencies. For each resampled dataset, an outer 5-fold Group k-Fold cross-validation was applied to estimate generalization performance on unseen subjects. Within each training fold, a secondary group-aware partitioning was performed to create an internal validation subset, ensuring that no subject appeared in both training and validation sets. This validation subset was used to monitor model fitting and mitigate overfitting during training.

Performance metrics, including accuracy, area under the ROC curve (AUC), and sensitivity, were computed on the held-out test folds and averaged across folds and bootstrap iterations. Accuracy measures the proportion of correctly classified examples among all cases, encompassing both positive and negative instances. Sensitivity assesses the proportion of actual positive cases correctly identified, reflecting the model's ability to reduce false negatives. The AUC is the probability that a model correctly ranks a randomly chosen positive case (e.g., fatigue) higher than a randomly chosen negative case. These metrics enabled a comprehensive evaluation of performance.

Accuracy=(TP+TN)/(TP+TN+FP+FN) (1)

Sensitivity=TP/(TP+FN) (2)

To quantify the uncertainty of the estimates, 95% confidence intervals were derived from the empirical distribution of bootstrap results. In addition, feature importance scores were extracted from each trained model and aggregated across bootstrap iterations to evaluate the stability and consistency of predictor contributions. This combined framework integrates group-aware validation, stochastic resampling, and model interpretability to provide a rigorous and unbiased assessment of fatigue detection performance.

## 3 Results

### 3.1 Statistical test

The study involved 40 participants (70% male, 30% female) from Universitas Indonesia, with a mean age of 21.3 ± 1.9 years and an average driving experience of 2 to 5 years, as determined from the screening questionnaire. Heart Rate Variability (HRV) parameters, including time-domain and frequency-domain measures, were calculated from standardized RR-interval data collected and processed during a driving simulation. Participants experienced two sleep-duration conditions: normal sleep (>7 h) and sleep-deprived (<4 h), as derived from the dataset. For each condition, participants completed four driving sessions. These sessions were treated as repeated measures within the same condition. Subjective measures, including the Karolinska Sleepiness Scale (KSS) and Rate of Fatigue (ROF), were also recorded across four sessions. After processing the RR interval data, HRV variables were averaged and summarized. A summary of HRV data by sleep condition is presented in Table 1.

**Table 1:** Summary of HRV features.

Features	Normal Sleep			
	1	2	3	4
Mean RR	781.14 ± 94.03	729.29 ± 71.80	711.28 ± 74.01	661.38 ± 79.06
SDNN	38.07 ± 54.64	36.39 ± 13.12	44.05 ± 18.55	65.18 ± 35.28
Mean HR	89.90 ± 9.94	84.39 ± 8.50	82.13 ± 8.61	76.19 ± 9.30
RMSSD	62.04 ± 36.44	42.42 ± 25.47	37.96 ± 26.77	31.37 ± 41.04
VLF	226.20 ± 160.01	150.89 ± 117.95	127.80 ± 112.99	167.83 ± 574.46
LF	3,491.91 ± 18,807.28	699.04 ± 501.20	1,179.34 ± 1,576.65	3,643/85 ± 10,014.34
HF	1,796.61 ± 1,869.13	898.70 ± 1,290.18	782.52 ± 1,314.68	717.50 ± 2,403.78
LF/HF	60.45 ± 371.27	3.95 ± 7.14	12.30 ± 42.61	124.13 ± 596.92

**Features**	**Sleep Deprived**			
	**1**	**2**	**3**	**4**
Mean RR	781.25 ± 63.22	736.02 ± 47.12	701.12 ± 22.60	682.38 ± 86.02
SDNN	54.13 ± 79.70	43.61 ± 27.58	44.80 ± 44.84	35.67 ± 13.60
Mean HR	90.84 ± 7.52	85.80 ± 5.61	81.65 ± 2.58	78.20 ± 11.91
RMSSD	80.20 ± 126.99	48.67 ± 62.20	30.91 ± 4.59	33.63 ± 42.88
VLF	1,487.64 ± 5,758.49	499.11 ± 2,301.85	99.65 ± 19.68	440.49 ± 2,244.30
LF	1,654.89 ± 3,964.59	2,018.52 ± 7,069.72	3,161.57 ± 14,685.53	761.35 ± 600.01
HF	4,447.12 ± 14,461.10	1,150.16 ± 3,242.83	372.54 ± 130.17	744.91 ± 2,349.76
LF/HF	6.03 ± 23.72	4.86 ± 14.17	11.25 ± 55.38	7.98 ± 10.29

Mean RR, Mean of RR intervals; SDNN, Standard deviation of the NN intervals; Mean HR, Mean of heart rate; RMSSD, Root means square of successive NN intervals differences; VLF, Very low frequency; LF, Low frequency; HF, High frequency; LF/HF, Low Frequency to High Frequency ratio.

Subjective data from the Karolinska Sleepiness Scale was collected using a scale ranging from 1 to 9 for each question, and data from the Rate of Fatigue was collected using a scale ranging from 0 to 10 for each question (Table 2).

**Table 2:** Summary of KSS and ROF.

Questionnaire	Normal Sleep			
	1	2	3	4
ROF	2.42 ± 1.66	3.32 ± 1.60	4.10 ± 1.80	4.70 ± 2.00
KSS	4.35 ± 1.62	5.05 ± 1.48	5.22 ± 1.85	5.50 ± 1.90

Questionnaire	Sleep Deprived			
	1	2	3	4
ROF	4.35 ± 2.11	5.40 ± 2.07	6.25 ± 2.19	6.70 ± 2.16
KSS	6.02 ± 1.51	6.72 ± 1.62	6.97 ± 1.35	6.97 ± 1.40

KSS, Karolinska Sleepiness Scale (measure of subjective sleepiness); ROF, Rate of Fatigue (measure of perceived fatigue).

The repeated-measures ANOVA revealed that driving duration had a stronger influence on several HRV parameters than sleep duration, with significant effects on mean RR, mean HR, RMSSD, HF, and ROF, indicating that prolonged driving was consistently associated with changes in autonomic regulation (Table 3). Participants with longer driving duration showed lower mean RR, mean HR, RMSSD, and HF, which were associated with increased fatigue levels. In contrast, sleep duration did not significantly affect most HRV indices, though it did show robust effects on subjective fatigue measures (ROF and KSS), suggesting that sleep loss primarily affected perceived fatigue rather than physiological variability. Under sleep-deprived conditions and during longer driving sessions, ROF and KSS scores increased significantly. Notably, the interaction effects were limited; only SDNN showed a significant interaction of sleep and driving duration.

**Table 3:** Summary of effects from repeated measures ANOVA (F-values).

Variables	Sleep duration	Driving Duration	Interaction
Mean RR	0.096	**36**.08**	0.398
SDNN	0.304	1.347	**4**.914*
Mean HR	0.745	**35**.60**	0.165
RMSSD	0.539	**7**.829**	0.610
VLF	3.285	1.459	1.108
LF	0.887	0.620	0.978
HF	1.088	**3**.640*	1.293
LF/HF	2.512	0.969	0.829
ROF	**61**.47**	**36**.70**	0.099
KSS	**58**.93**	**6**.455**	0.223

* *p* < 0.050.

** *p* < 0.010.

Bold values show significant value.

Furthermore, we examined the associations between HRV indices and subjective ratings of sleepiness and fatigue. A Spearman's rank correlation was conducted to evaluate the relationships between HRV, ROF, and KSS. The results indicate that several pairs of variables among HRV, ROF, and KSS show significant correlations; detailed results are presented in Table 4.

**Table 4:** Spearman correlation test results for HRV, ROF, and KSS data.

Variable	Correlations	ROF	KSS
Mean RR	Correlation Coefficient	−.158	−.100
	Sig. (2-tailed)	.005	.075
	N	320	320
SDNN	Correlation Coefficient	.056	.007
	Sig. (2-tailed)	.315	.895
	N	320	320
Mean HR	Correlation Coefficient	−.148**	−.087
	Sig. (2-tailed)	.008	.122
	N	320	320
RMSSD	Correlation Coefficient	−.162**	−.099
	Sig. (2-tailed)	.004	.076
	N	320	320
VLF	Correlation Coefficient	−.160**	−.098
	Sig. (2-tailed)	.004	.081
	N	320	320
LF	Correlation Coefficient	.041	.038
	Sig. (2-tailed)	.460	.498
	N	320	320
HF	Correlation Coefficient	−.156**	−.095
	Sig. (2-tailed)	.005	.090
	N	320	320
LF/HF	Correlation Coefficient	.174**	.116*
	Sig. (2-tailed)	.002	.038
	N	320	320

* *p* < 0.050.

** *p* < 0.010.

ROF, Rating of Fatigue; KSS, Karolinska Sleepiness Scale; Mean RR, Mean of RR intervals; SDNN, Standard deviation of NN intervals; Mean HR, Mean of heart rate; RMSSD, Root mean square of successive NN intervals differences; VLF, Very low frequency; LF, Low frequency; HF, High frequency; LF/HF, Low frequency to high frequency ratio.

Several pairs of variables between HRV, ROF, and KSS show significant correlations (Figure 3). Specifically, RMSSD shows correlations with ROF (*r* = −0.162, *p* = 0.004) and KSS (*r* = −0.099, *p* = 0.076). VLF also shows a significant correlation with ROF (*r* = −0.160, *p* = 0.004). Additionally, HF shows a significant correlation with ROF (*r* = −0.156, *p* = 0.005), and LF/HF shows a correlation with ROF (*r* = 0.174, *p* = 0.002). The strongest relationship was found between ROF and KSS (*r* = 0.589, *p* = 0.000), as expected, because both are subjective measures of fatigue. These results suggest several significant relationships between HRV, ROF, and KSS, although the strength of the correlations varies.

**Figure 4:**
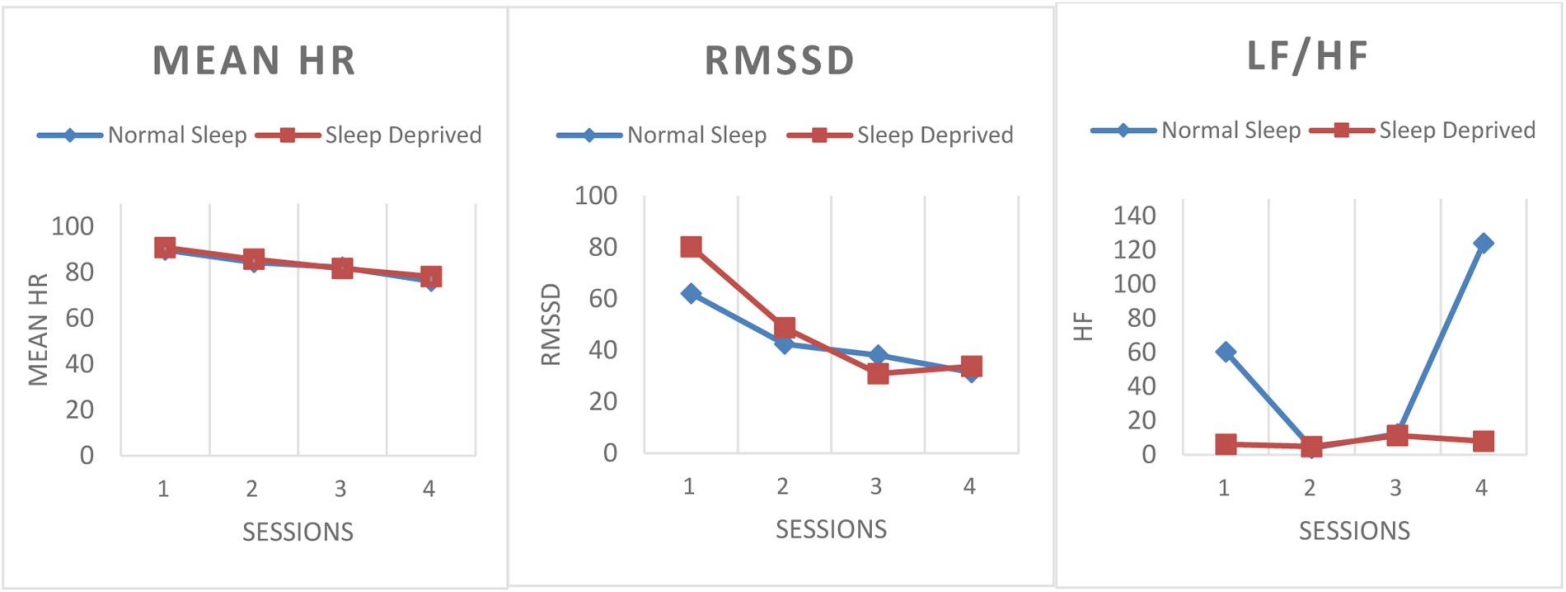
Session-wise trajectories of key HRV indices for normal-sleep and sleep-deprived conditions: **(a)** mean HR, **(b)** RMSSD, and **(c)** LF/HF ratio.

Figure 4 shows the session-wise trajectories of key HRV indices for normal sleep and sleep-deprived conditions: (a) Mean HR, (b) RMSSD, and (c) LF/HF ratio. In (a), both conditions showed a gradual decrease in Mean HR, indicating a reduction in heart rate over time, which is associated with increased drowsiness during the task. This trend is consistent with the negative correlation between Mean HR and ROF, in which increased fatigue is associated with lower heart rate. In (b), RMSSD, which reflects parasympathetic activity, declined across sessions for both conditions, with the sleep-deprived participants showing a more pronounced drop. This suggests a more substantial reduction in vagal tone due to sleep deprivation, which is supported by the negative correlation between RMSSD and ROF. Finally, (c) shows the LF/HF ratio, which exhibited the most significant divergence between conditions. The normal-sleep condition showed large fluctuations in LF/HF values, with a notable increase in Session 4, whereas the sleep-deprived condition had relatively stable, lower LF/HF values. The positive correlation between LF/HF and ROF suggests that higher LF/HF ratios, indicative of sympathetic dominance, are associated with greater fatigue.

### 3.2 Fatigue classifications

The classifiers used in the model were Logistic Regression and Ensemble Learning models, including Random Forest and XGBoost. The results indicate that fatigue associated with driving duration (DD) is more readily detectable than fatigue induced by sleep deprivation (SD). Across all classifiers, the DD models achieved higher accuracy, sensitivity, and AUC compared to SD models, suggesting that prolonged driving produces more distinct and consistent physiological changes (Table 5). In contrast, SD-related fatigue appears to be subtler and more variable across individuals, resulting in lower classification performance. Among the evaluated models, XGBoost demonstrated the best overall performance for both tasks, particularly for DD (AUC = 0.785, Sensitivity = 0.800), while incurring substantially lower computational cost (396.39 s) than Random Forest (1,508.18 s). Logistic Regression showed limited performance for SD, indicating that linear models are insufficient to capture the complex physiological patterns associated with sleep deprivation. These findings highlight the importance of considering the underlying fatigue mechanism when developing predictive models.

**Table 5:** Classification model performance evaluation.

Classifier	Factors	Accuracy	Sensitivity	AUC	Computation time
Logistic Regression	SD	0.567 (0.480–0.641)	0.621 (0.400–0.763)	0.615 (0.505–0.700)	53.53 s
	DD	0.751 (0.707–0.789)	0.788 (0.720–0.843)	0.751 (0.709–0.789)	60.70 s
Random Forest	SD	0.702 (0.626–0.763)	0.640 (0.534–0.736)	0.773 (0.701–0.831)	1,348.69 s
	DD	0.757 (0.706–0.803)	0.776 (0.710–0.836)	0.779 (0.731–0.824)	1,508.18 s
XG Boost	SD	0.708 (0.644–0.763)	0.619 (0.530–0.700)	0.761 (0.685–0.819)	392.12 s
	DD	**0.770** **(0.727–0.808)**	**0.800** **(0.738–0.850)**	**0.785** **(0.739–0.832)**	**396.39 s**

Classifier, The machine learning algorithm used (e.g., Logistic Regression, Ensemble Learning: Random Forest and XG Boost); SD, Sleep Duration; DD, Driving Duration; Accuracy (Testing), The percentage of correctly classified instances during the testing phase; Sensitivity, The ratio of true positive predictions to the actual number of positives; AUC, The probability that a model correctly ranks a randomly chosen positive case (e.g., fatigue) higher than a randomly chosen negative case; Computation Time, The time required to train and test the classifier (in seconds).

Bold values show significant value.

The results demonstrate that XGBoost outperforms Random Forest and Logistic Regression in detecting fatigue based on HRV features. This superiority can be attributed to XGBoost's ability to model complex nonlinear relationships and interactions among physiological variables. HRV signals are inherently nonlinear and influenced by multiple interacting autonomic processes. While Random Forest captures nonlinearities to some extent, its averaging mechanism limits its ability to model subtle decision boundaries. Logistic Regression, on the other hand, assumes linear relationships and is therefore less suited to capturing the complex dynamics of physiological signals. The use of subject-independent cross-validation ensures that the reported performance reflects true generalization to unseen individuals, which is critical for real-world deployment in fatigue monitoring systems.

The hyperparameter tuning process for the XGBoost model resulted in an optimal configuration with n_estimators = 159, learning rate = 0.0198, and max depth = 5. The relatively low learning rate indicates a gradual learning process, which contributes to improved model stability. Model complexity was controlled through min_child_weight = 4 and gamma = 0.0914, which restricts the creation of nodes that do not provide significant information gain, thereby reducing the risk of overfitting. In addition, subsampling strategies were applied using subsample = 0.7366 and colsample_bytree = 0.9425, allowing random sampling of observations and features during training to enhance model generalization. Overall, this configuration reflects an effective balance between model complexity and generalization performance.

**Figure 5:**
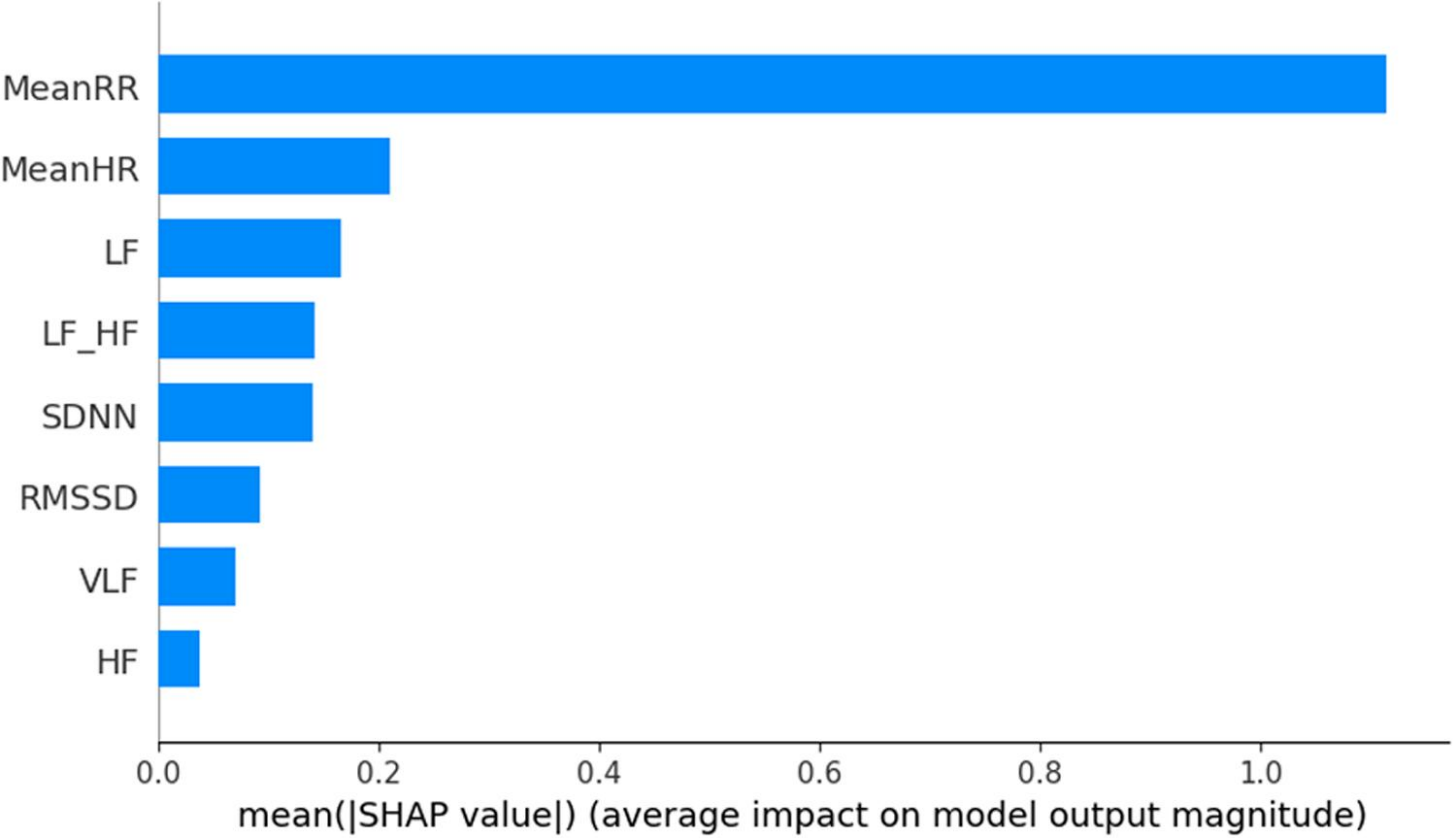
Feature importance based on mean SHAP values for driving duration. Mean HR, Mean of heart rate; SDNN, Standard deviation of the NN intervals; RMSSD, Root mean square of successive NN intervals differences; Mean RR, Mean of RR intervals; LF/HF, Low Frequency to High Frequency ratio; LF, low frequency; ROF, Rating of Fatigue; HF, High frequency.

In addition, we conducted a feature importance analysis to identify the most relevant features for predicting fatigue, using sleep duration and driving duration as classification variables. The feature importance of the XGBoost model was assessed using mean SHAP values, which quantify each feature's average contribution to the model's predictions. In previous driver fatigue studies, permutation-based and other feature selection methods were effectively used to identify relevant features for fatigue detection (48). SHAP values assess the impact of each feature on the model's output by attributing a contribution to each prediction. Figure 5 illustrates the mean SHAP values for each feature, with Mean RR showing the highest importance, indicating its strong influence on driving duration predictions. Other key features, such as Mean HR and LF, also played a significant role, whereas VLF and HF had lower importance, suggesting a relatively minor impact on the model's predictions.

**Figure 6:**
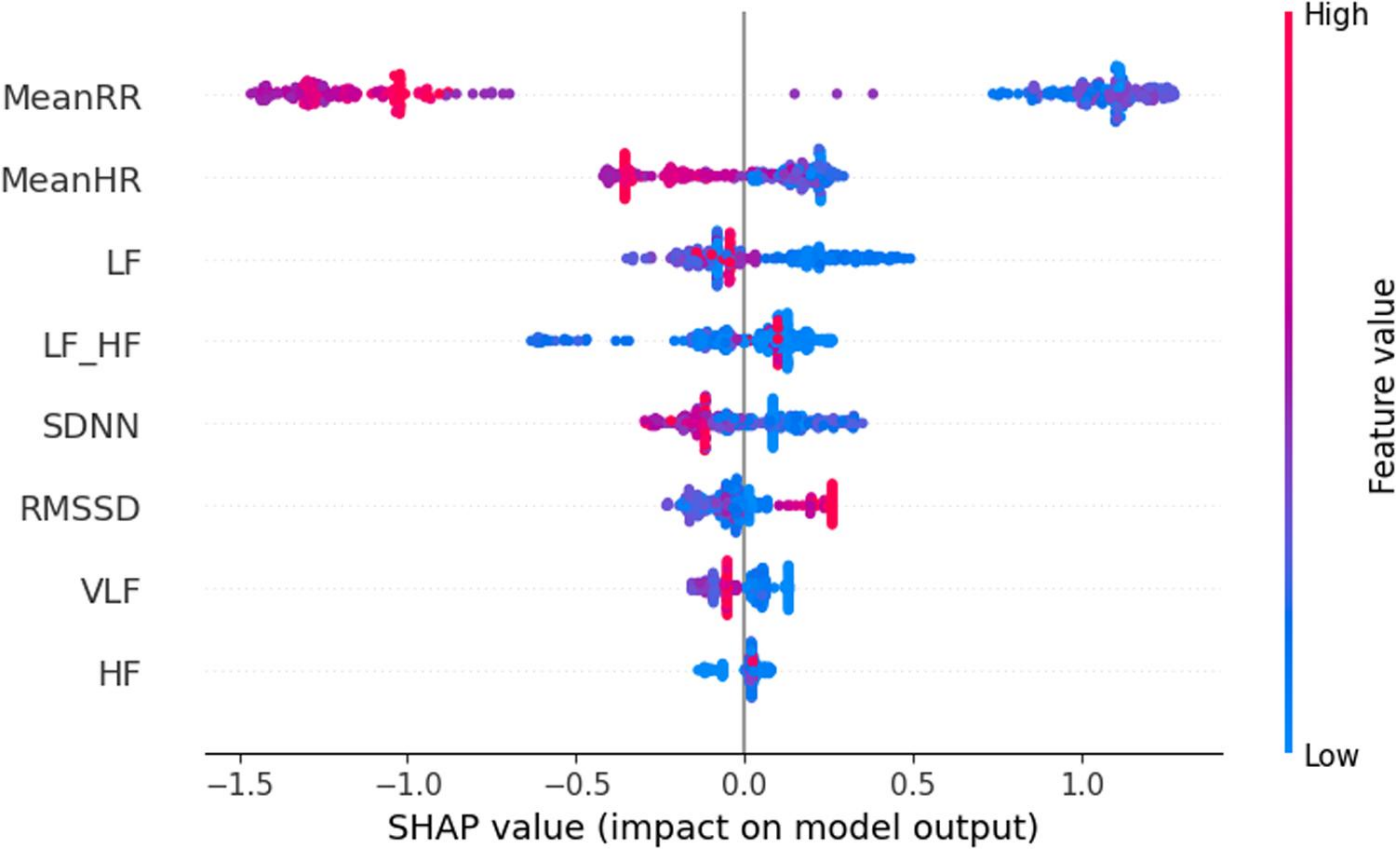
SHAP value distribution for XGBoost model features in driving duration. Mean HR, Mean of heart rate; SDNN, Standard deviation of the NN intervals; RMSSD, Root means square of successive NN intervals differences; Mean RR, Mean of RR intervals; LF_HF, Low Frequency to High Frequency ratio; LF, low frequency; HF, High frequency; SHAP, Shapley Additive Explanations (values indicating the contribution of each feature to the prediction); Feature Value, The actual value of the feature for an individual instance (used to calculate the SHAP value).

Figure 6 depicts the SHAP value distribution for each predictor, where the horizontal axis represents the direction and magnitude of the feature's contribution to the model output, and the color scale (blue, pink) encodes the feature value from low to high. In this model, the SHAP analysis indicates that fatigue associated with prolonged driving duration is characterized by reduced physiological arousal and altered autonomic regulation. Lower Mean HR values are strongly associated with an increased likelihood of fatigue, reflecting decreased cardiovascular activation, consistent with monotony-induced fatigue. Meanwhile, Mean RR contributes significantly to model predictions, suggesting sensitivity to subtle variations in cardiac timing dynamics. Reduced HRV metrics (SDNN and RMSSD) further indicate diminished autonomic flexibility. The LF/HF ratio increases with fatigue, reflecting autonomic imbalance rather than pure sympathetic dominance. Overall, these findings suggest that the model captures a complex interplay between reduced arousal and regulatory instability under prolonged driving conditions.

A *post hoc* power analysis was conducted using paired effect sizes (Cohen's d) to assess the study's sensitivity to detecting differences between short and long driving conditions (*N* = 40) (Table 6). The results revealed substantial variability in effect sizes across HRV features. Mean RR (*d* = −1.63) and Mean HR (*d* = −1.66) exhibited very large effect sizes with corresponding statistical power of 1.00, indicating a high likelihood of detecting true differences between conditions. RMSSD also demonstrated a moderate-to-large effect (*d* = −0.61) with high power (0.96), suggesting reliable detection of parasympathetic-related changes. In contrast, other HRV features such as SDNN (*d* = 0.19, powe*r* = 0.22), VLF (*d* = −0.23, powe*r* = 0.29), LF (*d* = 0.04, powe*r* = 0.06), HF (*d* = −0.36, powe*r* = 0.60), and LF/HF (*d* = 0.11, powe*r* = 0.11) showed small or negligible effect sizes with low statistical power, indicating limited sensitivity to detect differences for these variables. Overall, these findings suggest that while certain HRV features (particularly Mean RR, Mean HR, and RMSSD) exhibit robust differences between driving conditions, the study may be underpowered to detect smaller effects in other features.

**Table 6:** *Post-hoc* power analysis of driving duration.

Feature	Cohens_d	Power
MeanRR	−1.625671	1
SDNN	0.193936	0.223385
MeanHR	−1.660778	1
RMSSD	−0.610627	0.964482
VLF	−0.22825	0.290906
LF	0.039786	0.056932
HF	−0.358074	0.598217
LF_HF	0.111815	0.106058

It is important to note that *post hoc* power is inherently dependent on the observed effect size and sample size. Therefore, these results should be interpreted as descriptive of the current dataset rather than definitive evidence of adequacy or inadequacy of statistical power. Given the mathematical dependency between Mean RR and Mean HR, both variables exhibited similarly large effect sizes. However, their inverse relationship requires careful interpretation, and the results should be understood in the context of correlated physiological features rather than independent effects. The presence of large effect sizes in Mean RR and Mean HR, alongside weaker effects in other HRV features, suggests that fatigue induced by prolonged driving is primarily reflected in global cardiac dynamics rather than uniformly across all autonomic indices. This pattern further supports the notion that fatigue-related physiological changes are selectively expressed and may not be fully captured by individual HRV metrics in isolation.

## 4 Discussion

### 4.1 HRV and subjective fatigue responses across sleep and driving duration

The findings indicate that driving duration plays a more prominent role in shaping autonomic responses and perceived fatigue during simulated driving. Session-wise trajectories further validate these patterns. RMSSD declined progressively across Sessions 1–4 in both sleep conditions, but the decline was more pronounced under sleep deprivation, indicating that greater autonomic strain was associated with increased driving duration (34, 35). Mean HR also decreased across sessions, indicating greater parasympathetic activation. This was related to increased sleepiness among participants with long driving durations due to the monotonous task, who drove for 2 h. It is consistent with results stating that in chronic fatigue syndrome, lower heart rates were observed during nocturnal periods (36). Furthermore, time-on-task was associated with elevated subjective fatigue, decreased heart rate, and increased HRV, with the strongest associations in the vagal-mediated components (37). Previous studies have reported that sleep deprivation impairs cardiac autonomic function, characterized by an increase in the LF/HF ratio, reflecting sympathetic predominance and vagal withdrawal (38). However, in this study, the LF/HF ratio remained relatively low and stable. This pattern may indicate that prolonged sleep deprivation limits further autonomic modulation, resulting in reduced physiological responsiveness rather than continued increases in sympathetic indices. In contrast, the normal-sleep condition showed greater fluctuation, indicating more flexible autonomic responses.

Surprisingly, in the present study, sleep duration showed robust effects on subjective fatigue measures (ROF, KSS) but did not demonstrate significant effects on HRV parameters during the driving task. This finding contrasts with several studies showing that sleep deprivation reduces RMSSD and HF while increasing LF/HF ratio (38, 39). However, our results align with a study that found no changes in HRV following sleep deprivation under specific measurement conditions (40), and with research showing that sleep duration may have weaker associations with HRV than sleep quality (41). Some factors may explain this discrepancy. First, HRV was measured during active driving rather than at rest, and task engagement may have overridden sleep-related autonomic differences. Second, the lack of a significant effect of sleep duration on physiological measures in this study may be attributed to participants' age. Several studies have indicated that young, healthy, and physically active individuals often exhibit greater physiological resilience to acute sleep loss under specific conditions (40, 42).

Subjective measures aligned strongly with physiological changes. Participants in the sleep-deprived condition reported higher KSS (6.35) and ROF (5.19) scores than those in the normal-sleep condition (KSS = 4.70; ROF = 3.22), demonstrating heightened drowsiness and fatigue. Significant correlations between HRV parameters, especially RMSSD, VLF, HF, and Mean HR, and ROF further indicate that autonomic dysregulation directly influences subjective fatigue.

Taken together, these findings reveal that fatigue accumulates cumulatively with prolonged driving, and sleep restriction affects perceived fatigue rather than physiological fatigue. The convergence between HRV indices and subjective metrics supports a multimodal fatigue assessment approach rather than reliance on a single measurement type.

### 4.2 Fatigue detection modeling using machine learning

Machine learning analysis provided additional insights into how physiological and subjective indicators differentiate sleep conditions. This finding is also supported by Almutairi et al. (43), who found that combining physiological signals with deep learning algorithms improves classification. Among the tested models, XGBoost demonstrated the highest performance, achieving 77% accuracy and 80% sensitivity, surpassing both Logistic Regression and Random Forest. This superior performance reflects XGBoost's ability to model the nonlinear, high-dimensional patterns in HRV data that traditional linear models often fail to capture.

Feature importance analysis using SHAP values identified Mean RR as the most influential predictor, followed by mean HR, and LF, highlighting the dominance of time-domain HRV parameters in detecting physiological effects of sleep restriction. Lower Mean RR values were strongly associated with fatigue conditions. Fatigue causes the autonomic nervous system to shift toward sympathetic dominance, resulting in reduced variability in R-R intervals. This response is typically observed during physical exhaustion, mental fatigue, or stress (44, 45). It also affects the mean HR variable. The fatigue class showed a lower LF than the alert class, suggesting increased drowsiness among participants (46). In contrast, frequency-domain metrics (HF, LF/HF) and subjective variables (e.g., ROF) contributed minimally to prediction. The dominance of Mean RR, Mean HR, and LF reflects their close relationship with autonomic nervous system activity, where fatigue is characterized by increased sympathetic activation and reduced parasympathetic modulation. This aligns with sympathovagal imbalance theory and explains why time-domain HRV features are more sensitive indicators of fatigue than frequency-domain measures (21–23).

These results reinforce the viability of HRV-based machine learning models for automated fatigue detection. XGBoost's strong performance suggests that physiological signals contain distinctive nonlinear patterns indicative of fatigue, making it suitable for future real-time monitoring applications. Notably, the model's effectiveness underscores the strategic value of integrating HRV with supervised learning methods to address underreporting and misperception that are common in subjective assessments of driver fatigue.

### 4.3 Practical implications and limitations

Several limitations should be noted. First, a driving simulator may not fully replicate real-world driving conditions, such as unpredictable traffic, varied road textures, changing weather, or distractions, which can influence fatigue responses. Second, although the sample size (*n* = 40) met the statistical power requirements, it included only drivers from specific age groups, driving experience levels, or occupations, limiting the generalizability of the findings to the broader population.

Third, the study analyzed only HRV and subjective measures; other relevant fatigue indicators, such as reaction time, eye-blink rate, steering variability, or EEG signals, were not included. Including these multimodal inputs may strengthen both physiological interpretation and model accuracy. Fourth, individual differences in physical activity, emotional state, caffeine intake history, or sleep quality beyond duration may act as confounding variables that were not controlled in detail.

Integrating heart rate variability (HRV)-based fatigue detection with machine learning has practical implications for enhancing road safety. The reported 77% classification accuracy achieved by XGBoost using HRV parameters supports implementation through wearable PPG sensors or steering-wheel-embedded monitoring systems that transmit real-time alerts to drivers (47). This combination of physiological monitoring and machine learning enables transportation companies to proactively identify high-risk fatigue states before accidents, thereby transitioning from reactive, compliance-based strategies to predictive, data-driven fatigue management that improves driver safety and operational efficiency.

## 5 Conclusions

This study demonstrates that driving duration significantly influences both physiological and subjective fatigue responses in drivers; however, sleep duration affects perceived fatigue rather than physiological fatigue. Increased driving duration was associated with autonomic imbalance, as reflected in lower mean RR, mean HR, RMSSD, and HF, which were associated with increased fatigue levels. In contrast, sleep duration did not significantly affect most HRV indices, although it had robust effects on subjective fatigue measures (ROF and KSS). The correlation between the variables Mean HR, RMSSD, VLF, HF, and LF/HF and the ROF variable indicates that HRV parameters are associated with subjective fatigue indicators.

Machine learning results further showed that time-domain HRV measures, particularly Mean RR, Mean HR, and LF, emerged as the most influential predictors for sleep-related fatigue classification. These findings highlight the strong potential of HRV-based machine learning models for early fatigue detection, especially when drivers may underestimate or fail to report their declining alertness. These insights are derived from a machine learning model, XGBoost, which achieved the best performance among the tested classifiers, with 77% accuracy and the highest sensitivity.

Overall, integrating objective HRV metrics with subjective fatigue scales provides a robust multimodal approach to assessing driver fatigue. These insights can inform the development of real-time fatigue-monitoring systems to reduce fatigue-related risk in transportation. Future studies should incorporate more diverse populations, real-world driving contexts, and additional physiological and behavioral indicators to enhance model accuracy and generalizability. However, because this study was conducted in a controlled experimental setting without external validation or longitudinal testing, the proposed model should be considered a proof of concept rather than a fully deployable system. Future studies should incorporate more diverse populations, real-world driving contexts, and additional physiological and behavioral indicators to enhance model accuracy and generalizability. The practical implications of integrating heart rate variability (HRV)-based fatigue detection with machine-learning-supported implementation via wearable PPG sensors or steering-wheel-embedded monitoring systems that transmit real-time alerts to drivers. This combination of physiological monitoring and machine learning enables data-driven fatigue management, improving driver safety and operational efficiency.

## Data Availability

The original contributions presented in the study are included in the article/supplementary material, further inquiries can be directed to the corresponding author/s.
